# P-1190. Targeting Phage Defense Mechanisms: Expanding Phage Activity in the Context of Suspected abiF-Mediated Resistance against MRSA

**DOI:** 10.1093/ofid/ofaf695.1383

**Published:** 2026-01-11

**Authors:** Callan Bleick, Sean R Van Helden, Andrew D Berti, Michael J Rybak

**Affiliations:** Anti-Infective Research Laboratory, College of Pharmacy and Health Sciences, Wayne State University, Detroit, MI, Wayne State University School of Medicine, Department of Microbiology and Immunology, Detroit, MI, Detroit, Michigan; Wayne State University, Eugene Applebaum College of Pharmacy and Health Sciences, Detroit, Michigan; Wayne State University Colleges of Pharmacy and Medicine, Detroit, Michigan; Eugene Applebaum College of Pharmacy and Health Sciences, Detroit, Michigan

## Abstract

**Background:**

Phage resistance mechanisms represent a critical challenge in the advancement of phage therapy. *Staphylococcus aureus* utilizes multiple defense strategies, including adsorption inhibition via wall teichoic acid modification, restriction-modification systems, CRISPR-Cas systems, and abortive infection (Abi) pathways. The Abi system has been proposed to trigger bacterial self-destruction to prevent *Kayvirus* phage propagation, although its functional role in *S. aureus* remains poorly characterized.

**Figure:**
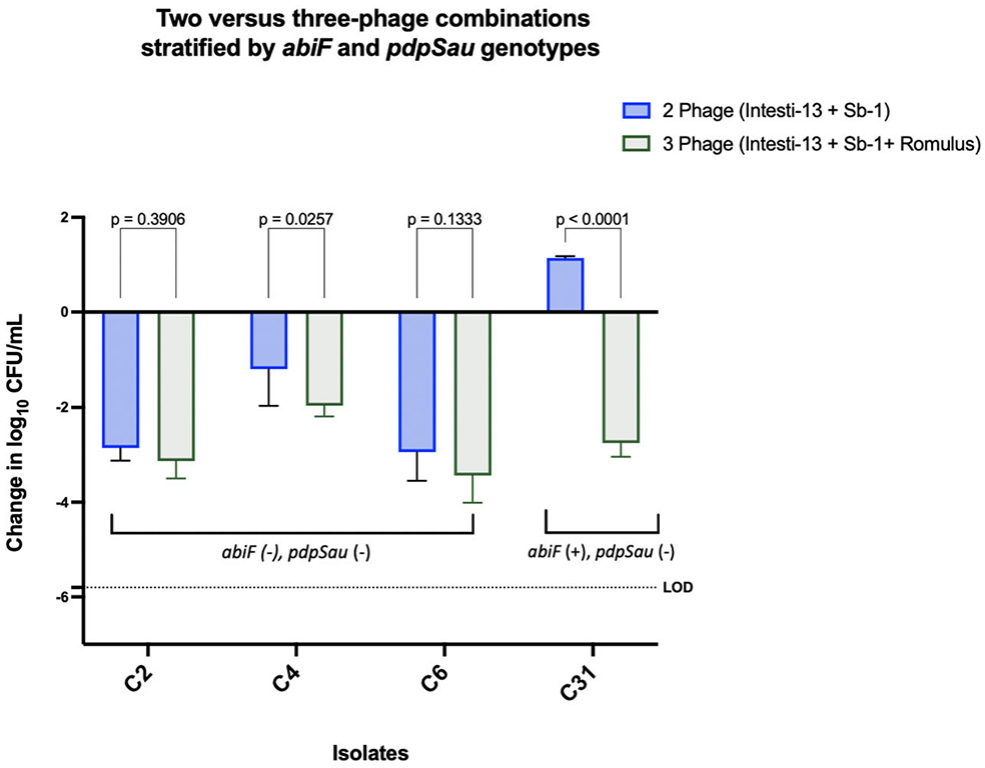
Two versus three-phage combinations stratified by abiF and pdpSau genotypes

**Methods:**

We evaluated four well-characterized daptomycin non-susceptible MRSA (DNS-MRSA) isolates from the Cubist repository. Time-kill assays (24h) were performed using either a two-phage cocktail (Intesti13 + Sb-1) or a three-phage cocktail (Intesti13 + Sb-1 + Romulus) at a multiplicity of infection (MOI) of 1.0. Change in bacterial burden was measured as log_10_ CFU/mL reduction. Statistical analysis was conducted using two-way ANOVA with Tukey's post hoc test (p < 0.05). Whole genome sequences were used to assess the presence of known phage defense systems.

**Results:**

While the two-phage cocktail modestly reduced bacterial burden in most DNS-MRSA isolates, adding Romulus significantly enhanced killing in select strains. The most pronounced effect was in C31, which harbors the *abiF* gene; this isolate was resistant to the two-phage cocktail (+1.14 ± 0.04 log_10_ CFU/mL) but exhibited a > 4.0 log_10_ reduction with the three-phage combination (–2.75 ± 0.29, *p* < 0.0001), suggesting Romulus may evade *abiF*-mediated defense. In contrast, *abiF*-negative isolates (C2, C4, C6) responded similarly to both cocktails, indicating a potential K-phage resistance mechanism.

**Conclusion:**

These findings suggest that *abiF* may mediate resistance specific to *Kayirus*-like phages, with *Silviavirus*-like phages remaining unaffected. Incorporating phages with distinct infection mechanisms may help bypass such resistance and enhance therapeutic efficacy. A diverse phage cocktail could be key to overcoming intrinsic defenses and optimizing empiric treatment for multidrug-resistant *S. aureus.*

**Disclosures:**

Michael J. Rybak, PharmD, PhD, MPH, Abbvie: Grant/Research Support|Innoviva: Grant/Research Support|Melina: Grant/Research Support|Merck: Grant/Research Support|Shionogi: Grant/Research Support

